# Adapting High-Resolution Respirometry to Glucose-Limited Steady State Mycelium of the Filamentous Fungus *Penicillium ochrochloron*: Method Development and Standardisation

**DOI:** 10.1371/journal.pone.0146878

**Published:** 2016-01-15

**Authors:** Christoph W. Schinagl, Pamela Vrabl, Wolfgang Burgstaller

**Affiliations:** University of Innsbruck, Institute of Microbiology, Innsbruck, Austria; Universidad de La Laguna, SPAIN

## Abstract

Fungal electron transport systems (ETS) are branched, involving alternative NADH dehydrogenases and an alternative terminal oxidase. These alternative respiratory enzymes were reported to play a role in pathogenesis, production of antibiotics and excretion of organic acids. The activity of these alternative respiratory enzymes strongly depends on environmental conditions. Functional analysis of fungal ETS under highly standardised conditions for cultivation, sample processing and respirometric assay are still lacking. We developed a highly standardised protocol to explore *in vivo* the ETS—and in particular the alternative oxidase—in *Penicillium ochrochloron*. This included cultivation in glucose-limited chemostat (to achieve a defined and reproducible physiological state), direct transfer without any manipulation of a broth sample to the respirometer (to maintain the physiological state in the respirometer as close as possible to that in the chemostat), and high-resolution respirometry (small sample volume and high measuring accuracy). This protocol was aimed at avoiding any changes in the physiological phenotype due to the high phenotypic plasticity of filamentous fungi. A stable oxygen consumption (< 5% change in 20 minutes) was only possible with glucose limited chemostat mycelium and a direct transfer of a broth sample into the respirometer. Steady state respiration was 29% below its maximum respiratory capacity. Additionally to a rotenone-sensitive complex I and most probably a functioning complex III, the ETS of *P*. *ochrochloron* also contained a cyanide-sensitive terminal oxidase (complex IV). Activity of alternative oxidase was present constitutively. The degree of inhibition strongly depended on the sequence of inhibitor addition. This suggested, as postulated for plants, that the alternative terminal oxidase was in dynamic equilibrium with complex IV—independent of the rate of electron flux. This means that the onset of activity does not depend on a complete saturation or inhibition of the cytochrome pathway.

## Introduction

**Fig 1:**
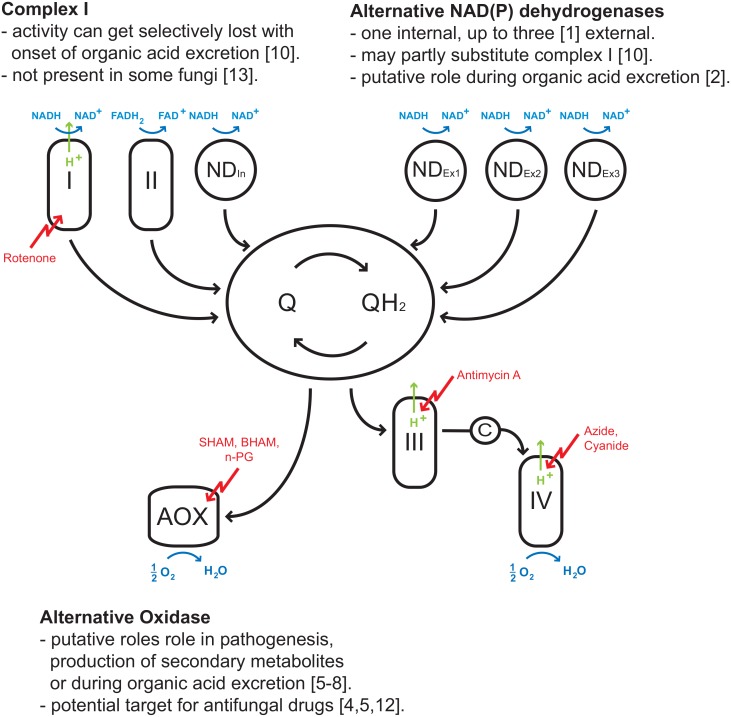
Multiple convergent pathways for electrons into the quinone pool of fungal mitochondria. Electrons flowing downstream the quinone pool are drained into the cytochrome pathway (complex III and IV) or via an alternative route to AOX. Arrows indicate the direction of electron fluxes downstream the thermodynamic cascade of the ETS.

The inner membrane of fungal mitochondria may contain—beside the standard components (complexes I, II, III, IV) of the electron transport system (ETS)–several alternative redox enzymes (Fig 1): (i) up to three alternative external NAD(P)H dehydrogenases [1] or (ii) an alternative internal NAD(P)H dehydrogenase [2], both of which feed electrons additionally to complex I into the quinone pool. An (iii) alternative oxidase (AOX), works in parallel to the cytochrome c oxidase (COX) by reducing oxygen to water. These alternative redox enzymes have been suggested to be involved in many metabolic activities. For instance, the AOX is reported to be induced by a functionally compromised COX in *Neurospora crassa* [3, 4], or being involved in the pathogenesis of *Aspergillus fumigatus* [5] or active during the production of cephalosporin C by *Acremonium chrysogenum* [6]. In *A*. *niger* the AOX is considered to serve as an electron overflow valve during excretion of citrate [7, 8]. As the P/O ratio is significantly decreased with this alternative respiration, it enables the fungus to cope with conditions of energy excess by maintaining the essential NADH reoxidation [9, 10]. In addition, the fungal complex III and the AOX have been targets for fungal pest management in agriculture [11]. Even more, the AOX has also come into focus as a potential drug target of pathogenic fungi in humans [4, 5, 12].

Per definition, the AOX activity is reflected in the respiration which (i) remains after inhibiting COX and (ii) is sensible to inhibitors of AOX, mostly hydroxamic acids. This definition determines the sequence of inhibitor application. The classical tool to functionally study the ETS is a respirometric assay, i. e. a titration protocol for specific substrates, inhibitors and uncouplers [13].

Especially for filamentous fungi, this approach bears some challenges. One problem is that filamentous fungi are multi-septate organisms with an inhomogeneous distribution of mitochondria which leads to a high variability in oxygen consumption rates [14]. A second problem is that the combined barriers of cell wall and plasma membrane [15] may hinder substrates and inhibitors to permeate into the hyphae. Thirdly, multidrug efflux pumps may remove cytotoxic substances from the cytoplasm [16]. And fourth and foremost, fungi exhibit an enormous phenotypic plasticity and adjust to slightest changes in environmental conditions with alterations in physiology (e. g. activity of respiratory complexes) and morphology [17, 18]. This phenotypic plasticity forces the experimenter to highly standardise margin conditions for cultivation, sample processing and the respirometric assay. In addition, recent research in several fields of microbial physiology such as metabolomics indicates that the development of physiological methods applicable to many organisms is not possible or meaningful [19, 20].

There is increasing awareness that it is necessary to critically revise and adapt methods for each organism anew [19, 21, 22] and to work with physiologically well-defined cultures [23]. Nevertheless, because of a lacking standard protocol and a great variety of applied cultivation and sample processing methods (Table 1), the comparison of literature data is still critical within this field of research. It was, therefore, one of our aims to exemplify how extensive and difficult the standardisation of such a method may be and that overall generality for such a method is not possible.

**Table 1:** Compilation of studies dealing with the ETS of filamentous fungi by applying inhibitors. Cultivation conditions, sampling time, sample preparation and respirometric assay conditions are given.n.a., not available;

Organism	Cultivation			Sample preparation		Assay conditions			Ref.
	Cultivation in	T (°C)	pH	Sampling time	Sample preparation	Assay medium	T (°C)	pH	
*Phycomyces blakesleeanus wt* (Burgeff) (NRRL 1555(-))	Petri dish	20	4.5	28 d	Aeration for 10 min. of broth diluted with fresh nutrient medium	Broth with supplemented fresh minimal medium	25	4.5	[24]
*Acremonium chrysogenum* ACC 46117	Shake flask	28	n.m.	time course over 70 h	1 mL culture broth	1 mL culture broth	28	n.a.	[6]
*Aspergillus niger* B60	Bioreactor	30	Start 3.0	60 h	2 mL culture broth withdrawn and immediately processed	Culture broth	n.a.	n.a.	[7]
*Aspergillus niger* WU-2223L	Sakaguchi shake flask	30	Start 3.0	Late exponential phase, 5 d	n.a.	0.5 mM glucose, 10 mM K phosphate buffer,	30	7.2	[25]
*Aspergillus niger* WU-2223L	Sakaguchi shake flask	30	Start 3.0	1 d	Broth filtered 1.6 μm	8% (w/v) glucose, 10 mM K phosphate buffer	30	7.0	[26]
*Magnaporthe grisea* wt	Erlenmeyer shake flask	28	n.a.	1 d	Centrifuged, washed 3 x with 20 mM Hepes-Tris buffer (pH 7)	20 mM Hepes-Tris buffer	25	7.0	[27]
*Metarhizium anisopliae* ARSEF 2038	n.a.	27	n.a.	10 d	Broth filtered 0.45 μm, washed with 2 volumes of distilled sterile water	Salt solution with 1% (m/v) glucose	25	n.a.	[28]
*Neurospora crassa* NCN 235	n.a.	27	n.a.	6 d	Broth filtered 0.45 μm, washed with 2 volumes of distilled sterile water	Salt solution with1% (m/v) glucose	25	n.a.	[28]
*Neurospora crassa* wt RL21a	Florence flask	25	5.8	Mid exponential phase	Broth filtered 1.2 μm	Vogels minimal medium + 2% glucose	25	5.8	[29]
*Acremonium chrysogenum* W53.2.53	Chemostat	28	6.6	n.a.	n.m.	n.m.	28	n.a.	[30]
*Aspergillus niger B1-D*	Glucose-limited chemostat	25	4.0	n.a.	1–2 mL culture broth filtered 1.6 μm	Sterilized culture medium	27	4.0	[31]

The main aim of this work was to establish a titration protocol for inhibitors of both standard and alternative terminal oxidase as well as for uncoupling agents of the mitochondrial proton gradient in *Penicillium ochrochloron*. To achieve this, we strived for a high degree of experimental standardisation. This included the use of chemostat cultivated mycelium to ensure a well-defined and reproducible physiological state of hyphae. To disturb the steady state of the culture as little as possible by sampling, we opted for high-resolution respirometry because of its high accuracy with a minimum amount of sample.

## Materials and Methods

### 2.1 Chemicals

All chemicals for the respirometric assays were of analytical grade. Salicylhydroxamic acid (SHAM), benzhydroxamic acid (BHAM), carbonyl cyanide 3-chlorophenylhydrazone (CCCP), carbonyl cyanide 4-(trifluoromethoxy) phenylhydrazone (FCCP), 2,4-dinitrophenol (DNP), antimycin A and rotenone were purchased from Sigma-Aldrich and *n*-propyl gallate (n-PG) from Fluka. Potassium cyanide and sodium azide were supplied from Merck (VWR) and N-2-hydroxyethylpiperazine-N′-2-ethanesulfonic acid (HEPES) from Roth. Stock solutions were prepared with glass distilled deionized water or absolute ethanol, respectively. Aqueous solutions of potassium azide and sodium cyanide were buffered with 10 mM HEPES-NaOH to pH 7.0. Solutions of SHAM, BHAM and azide were prepared freshly on the day needed and stock solutions with absolute ethanol were handled in glass vials. All stock solutions were kept on ice during measurements and were extra protected from light for storage at -20°C. The anti-foam agent Clerol FBA 5075 (Cognis, Meaux, France) was used for the chemostat medium.

### 2.2 Organism

*Penicillium ochrochloron* CBS 123824 has developed from a wild type isolate (CBS 123823) after 20 years of passaging on rye. The wild type strain originated from a soil which was strongly contaminated with heavy metals and initially was identified as the closely related species *Penicillium simplicissimum* [32]. For both strains a re-identification revealed *Penicillium ochrochloron* as the correct species [33].

### 2.3 Media

The **preculture medium** consisted of (mM): (NH_4_)_2_SO_4_ 6.25, NH_4_Cl 12.5, KH_2_PO_4_ 5.8, MgSO_4_ 7H_2_O 1.6 and glucose⋅1H_2_O 400. The solution of 1 M HEPES-NaOH was adjusted to pH 7.3 at 30°C. Solutions of salts, glucose and buffer were autoclaved separately and mixed. For each liter of medium 10 mL of double filtered (0.2 μm cellulose acetate) trace element solution were added. The trace element solution consisted of (mM): Fe (II) SO_4_ 7H_2_O 3.59, Mn (II) SO_4_ 1H_2_O 2.72, ZnCl_2_ 2.935, Cu (II) SO_4_ 5H_2_O 0.40 and CaCl_2_ 2H_2_O 4.08.

The **chemostat medium** for glucose limitation [33] was basically a preculture medium without HEPES buffer and with the following modifications. The glucose concentration was reduced to 20 mM and (NH_4_)_2_SO_4_ (final concentration 12.5 mM) served as sole source for ammonia. Anti-foam agent was added to a final concentration of 0.1 g⋅L^-1^.

#### Respiration medium

Normally 10 minutes before starting the respirometric assay culture broth was sampled from the chemostat and immediately twice syringe-filtered (cellulose acetate, pore size 0.45 μm) to eliminate fat and oil droplets from the anti-foam agent and all biomass.

### 2.4 Cultivation

#### Preculture and inoculation of the bioreactors

To ensure a filamentous inoculum, *P*. *ochrochloron* was cultivated in the preculture medium which provided a high osmolality. A 500 mL Erlenmeyer flask containing 100 mL of medium was inoculated with spores to a final concentration of 10^6^−10^7^ mL^-1^ and shaken for 72 ±1 hours on an orbital shaker (25 mm amplitude) at 350 rpm, 30°C and a relative air humidity of 60%. The incubation chamber was kept dark for 95% of the cultivation time. Cultures left exponential growth phase when ammonium was depleted about 50 hours after inoculation.

#### Chemostat cultivation

In brief, chemostat cultivation was performed in a Biostat M bioreactor (Braun, Melsungen, Germany) with a working volume of 1.8 L as previously described [33]. Constant culture volume was maintained via a lateral glass pipe at the border between fluid and gas phase. Conditions were 30°C, 0.56 vvm dried air and 900 rpm, i.e. 2.12 m s^-1^ tip speed (three 4-bladed Rushton turbines mounted equidistant on the stirrer shaft). The pH was regulated to 7.0 with sterile 0.2 M NaOH. Two hours after inoculation the feed pump was started (3 mL min^-1^, D = 0.1 h^-1^). For reaching steady state conditions the cultures were allowed to pass through four hydraulic residence times (= 40 h). Steady state conditions were assumed if oxygen consumption, evolution of carbon dioxide and the formation of biomass stabilized. The morphology of steady state samples was monitored with light microscopy (phase contrast, magnification 500 to 1200 times with oil immersion). Immediately before every sampling of the steady state cultures 6 mL of broth was withdrawn and discarded.

Taken together, the changes to our previous procedures [33, 34] were (i) standardising the incubation time of the preculture, (ii) shortening the batch phase of the chemostat cultures and (iii) reducing the concentration of antifoam agent. The latter was necessary since higher concentrations of anti-foam negatively interfered with high-resolution respirometry (see section 3.1.2, Respiration medium). The previously used antifoam concentration of 1.0 g L^-1^ medium for glucose-limited conditions was reduced to 0.25 and finally to 0.1 g L^-1^. Despite the reduction of 90%, this concentration prevented formation of foam, if the feed medium in the reservoir was stirred constantly.

### 2.5 Method development sample preparation

During method development, several sample preparation methods were tested, of which the first three involved different types of filtration. The final method used a direct transfer of native culture broth into the respirometer. For details to each preparation method see S1 Appendix Sample preparation.

### 2.6 High-resolution respirometry

All respirometric assays were conducted with a high-resolution respirometer (Oxygraph-2k, Oroboros Instruments, Innsbruck, Austria). The corresponding software (DatLab 4.0, Oroboros Instruments, Innsbruck, Austria) was also used for analysis of data. The required stability of oxygen consumption rate was defined as a maximal decrease of 5% over a period of 20 minutes.

#### Optimization of the assay conditions

Assay conditions were fine tuned to the sample preparation method. This included the amount of biomass in the assay, the kind of respiration medium, the optimum agitation speed (varied from 300 to 700 rpm) and as well as the type and the concentration of inhibitors and uncouplers. As inhibitors for the AOX both hydroxamic acids SHAM (2.5 mM final concentration) and BHAM (2.5 mM final concentration) and also *n*-propyl gallate (n-PG; 2–3.5 mM final concentration) were tested. Cyanide (1.0 mM final concentration) and azide (0.7 mM final concentration) were used for inhibition of COX. For uncoupling of the ETS the protonophors CCCP (2 μM final concentration), FCCP (1 μM final concentration) and DNP (2 μM final concentration) were tested. Determination of the optimum concentrations for inhibitors and uncouplers were performed by a stepwise titration to maximal effect on oxygen consumption rates.

#### Optimized method and standardised sequence of assays

**Fig 2:**
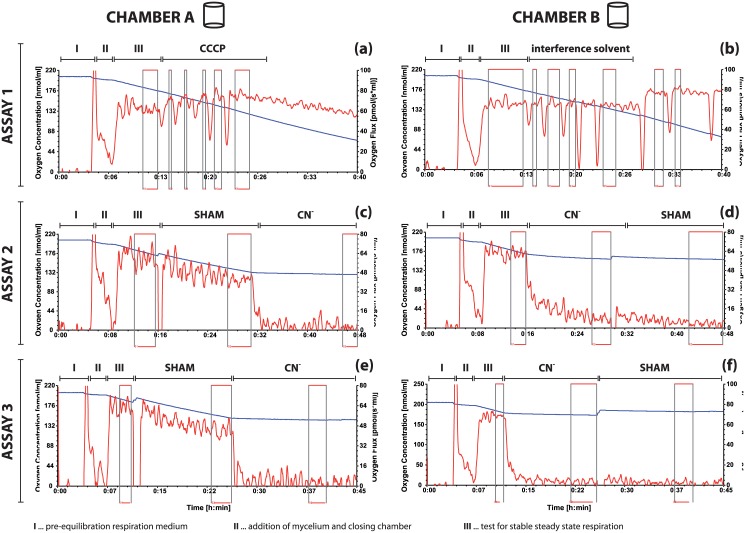
Representative original respirometer traces illustrating the standardised respirometric assay with glucose-limited steady state mycelium of *Penicillium ochrochloron*. For the assays steady state mycelium was used and resuspended in the respiration medium. The assay consisted in total of six single measurements in three runs with two chambers, respectively (blue line, y_1_ axis: oxygen concentration; red line, y_2_ axis: oxygen consumption rate). Marked sections (red boxes) indicate data range for the calculation of averaged fluxes. (a and b) Assay 1: effect of uncoupler and solvent. (c and d) Assay 2: effect of SHAM and cyanide, with either SHAM prior to or after cyanide. (e and f) Assay 3: identical to assay 2, but 24 hours later for validating reproducibility and monitoring the stability of chemostat culture.

Prior to the respirometric assay, each chamber of the respirometer was filled with 2 mL respiration medium. With both stoppers partly inserted, the respiration medium was stirred at 300 rpm for 4–5 minutes to equilibrate with the atmospheric oxygen and to stabilize the temperature at 30°C (Fig 2a–2f, phase I). Afterwards to each chamber three droplets of chemostat broth were directly transferred (see Fig A d in S1 Appendix Sample preparation). As soon as the flux signal settled (Fig 2a–2f; Phase II) chambers were immediately closed.

In total, a sequence of three standardised assays (Fig 2) was performed. In the first assay, the stability of the oxygen consumption was checked (Fig 2a and 2b; phase III) before in one of the chambers the maximal uncoupling capacity of the ETS was determined. This was done with a gastight syringe and by a stepwise (1 μL) titration of 1 mM CCCP stock solution (Fig 2a, CCCP) to maximal effect. To test for interferences of the solvents with the oxygen consumption rate an equal volume of solvent was applied within the same time course to the second chamber (Fig 2b; interference solvents). The amount of organic solvents never exceeded 1% of the assay volume, i.e. 20 μL.

In the subsequent second assay (Fig 2c and 2d) both terminal oxidases were treated after the oxygen consumption rate had become stable (Fig 2c and 2d; phase III) with a *vice versa* sequence of inhibitors: adding 20 μL 250 mM SHAM followed by 20 μL of 100 mM cyanide (Fig 2c; SHAM, CN^-^), reversed sequence in the second chamber of respirometer (Fig 2d, CN^-^, SHAM).

In the third assay, which was performed approximately 24 hours later, both terminal oxidases were again inhibited exactly as in the second assay (Fig 2e and 2f).

Before and after each assay chambers and stoppers were rinsed several times with double distilled water. The subsequent cleaning with 70% ethanol and ethanol absolute was performed for each step at least 10 to 15 minutes or overnight when possible.

#### Normalization of oxygen consumption rates

As a consequence of the small amount of biomass in the respirometric assay a gravimetric estimation of dry weight was not possible in a meaningful way (see Biomass concentration in section 3.1.2 and S2 Appendix Biomass). Therefore oxygen consumption rates resulting from the respirometric assay were normalized as follows: corresponding steady state oxygen consumption rates were defined as 100% and rates were expressed relative to this value.

To crosscheck the results from the few reliable gravimetric estimations (i.e. c. 0.2 (mg DW) per chamber) we back calculated the biomass in the respirometric assay from the concentration of biomass in the chemostat cultivations. The goal was to check, if the order of magnitude equals for both approaches. Details are given in S2 Appendix Biomass.

## Results

### 3.1 Method development

The foremost goal was to preserve the physiological state of samples during handling and the respirometric assay. Unfortunately, *P*. *ochrochloron* [33] and filamentous fungi in general [18] exhibit a high sensitivity of physiological processes to very small changes in margin conditions (i. e. physiological phenotypic plasticity). Therefore, to achieve our goal, a rigorous standardisation was necessary on three levels: cultivation (preculture, growth in chemostat), sample preparation (method to transfer mycelium into the respirometer), and respirometric assay (assay medium, agitation speed, biomass concentration, kind of inhibitors or uncouplers and the sequence of addition).

#### 3.1.1 Standardisation of cultivation and sample preparation

Concerning cultivation, neither mycelium from shake flask cultures, nor from bioreactor batch cultures (early, mid or late exponential phase) exhibited the necessary stability of respiration rate as defined in Materials and Methods (maximal decrease of 5% over a period of 20 minutes). In contrast, mycelium from glucose limited chemostat cultivation (with slight modifications to our previous standard procedure, see 2.4) showed a sufficiently stable oxygen consumption rate. We therefore used solely mycelium from glucose-limited chemostat for all further steps of method development.

Optimization of sample preparation was started (Fig A a in S1 Appendix Sample preparation) with a mild filtration of the culture broth (gravitation, mesh size 0.5 mm; duration approximately two minutes). The mesh size was chosen to collect only dispersed hyphae in the filtrate and to withhold the small portion of hyphal aggregates, which formed inevitably during cultivation. The amount of biomass collected in the filtrate was, however, too low to generate a measurable signal for oxygen consumption. Neither increasing the filtrated broth volume, nor a subsequent mild vacuum filtration (maximal 250 mbar below atmospheric pressure; Fig A b* in S1 Appendix Sample preparation), nor a fastened filtration by using pressure (60 seconds; Fig A c in S1 Appendix Sample preparation) provided reproducible oxygen consumption rates.

To avoid any potential negative effects of washing steps and filtration we thus transferred a broth aliquot directly from a sample from the bioreactor into the respirometer using cut pipette tips to widen the tip opening (Fig A d in S1 Appendix Sample preparation). This sample handling had the disadvantage of transferring antifoam agent, but this problem could be kept at a minimum due to the small transferred volume and the reduced antifoam concentration in the chemostat medium (section 2.4). Another drawback was that the sample contained also a small proportion of mycelial aggregates, which made it difficult to control the amount of biomass in the assay. These uneven amounts were reflected in different levels of oxygen consumption rates. Despite of these drawbacks, this procedure resulted in a reproducible and stable steady state respiration rate (Fig 2a–2f, phase III) and shortened the time for sample preparation to 30 seconds.

#### 3.1.2 Standardisation of the respirometric assay

The conditions of the respirometric assay had to be adjusted on the level of biomass concentration, agitation speed and respiration medium as well as kind and concentration of inhibitors and uncouplers.

Biomass concentration. For adjusting the optimal biomass concentration we had to meet partly opposing demands. (i) The necessary minimum time required for the titration protocol, given by the time needed for an inhibitor to permeate the hyphal cell wall and plasma membrane. (ii) The amount of available oxygen in the closed chamber of the respirometer: The biomass concentration had to be low enough to allow the performance of the whole titration protocol before the oxygen concentration dropped to a critical level. (iii) The oxygen consumption signal had to be sufficiently high to enable a reliable discrimination of an inhibitor effect. (iv) The oxygen consumption rate had to be adequately low to allow the measurement of elevated fluxes from the treatment with uncouplers.

Taken together the above mentioned issues demanded to use of 2–3 drops of culture broth per chamber. To estimate how much dry weight this volume corresponded to, a calculation was done which resulted in an amount of 0.27 (mg DW) 2 mL^-1^ (2.6, Normalization of oxygen consumption rates and S2 Appendix Biomass). This value is only for orientation how much biomass a chamber approximately contained during a respirometric assay.

Normalization of oxygen consumption rates. The slightly inhomogeneous distribution of biomass in the sample caused an uneven concentration between both measuring chambers. This suggested harvesting the biomass quantitatively after finishing the respirometric assay. It was, however, not possible to win the complete biomass from the chamber and to determine this amount of biomass by weighing.

The use of other markers for the normalization of the oxygen consumption rate would have had the prerequisite of winning all biomass from the chamber of the respirometer. This was not possible or would—with high probability—have introduced additional errors (e g. because of the necessary cell permeabilisation or cell disruption).

We therefore defined the steady state oxygen consumption rates (biomass without inhibitors) in the respirometer as 100% and other rates were expressed relative to this value. Because this normalization was a straightforward, non-invasive procedure without introducing additional errors, we decided to do it that way.

Agitation speed. A higher stirrer speed is accompanied by a lower noise of the oxygen consumption signal. On the other hand it is well-known that filamentous fungi are sensitive towards shear forces [35]. Despite significantly decreased tip speeds in the high-resolution respirometer (0.235 m⋅s^-1^ at 300 rpm to 0.548 m⋅s^-1^ at 700 rpm) compared to that in the bioreactor (2.12 m⋅s^-1^), the different geometries of the vessels and type of stirring (Rushton turbines versus magnetic stirrer bar) made it necessary to monitor the impact of the agitation on signal stability and mechanical integrity of the mycelium over a period of 20 minutes. Therefore mycelium was sampled before and after agitation in the respirometer and checked by light microscopy for any changes in morphology (i.e. signs of disintegration like broken off branches or hyphae depleted of cytoplasm). The demand of hyphal integrity and minimal signal noise could only be met at a comparably low stirrer speed of 300 rpm.

Respiration medium. Previous studies with *P*. *ochrochloron* indicated that the choice of assay medium has a strong impact on the results of physiological experiments [33]. In this work, the respiration medium was gained by double filtration of a broth aliquot from a glucose-limited steady state culture. Due to the buffer effect of the phosphate in the chemostat medium, it was not necessary to add a buffer to keep the pH constant during the assay. This provided a respiration medium which was similar to the actual chemostat conditions but without oil or fat from the anti-foam agent. The oil and fat fraction of the anti-foam agent generated a thin smear on the surface of the measuring chambers after several assay. This diminished the diffusion properties of the polarographic sensor membranes or served as a reservoir for lipophilic substances.

To check if it was necessary to supplement the respiration medium (i.e. the double filtered glucose-limited broth which contained almost no residual glucose) with the limiting nutrient glucose or not, we compared the oxygen consumption rates under both conditions. Interestingly, when glucose was supplemented, the oxygen consumption rates became unstable, while oxygen consumptions rates in non-supplemented samples remained stable (for original traces see S1 Fig. Glucose supplement).

Inhibitors and uncouplers. To test whether or not complex I or III were functional in *P*. *ochrochloron* during glucose limited growth we checked the effect of rotenone (final concentration 3 μM) and antimycin A (final concentration 20 μM). With rotenone we observed an immediate drop of oxygen consumption rate in the range of one third to one half of the steady state oxygen consumption rate. Antimycin A, an inhibitor for complex III, had no effect on the oxygen consumption rate at all (see S5 Fig. Rotenone Antimycin A).

Cyanide and azide were tested for inhibition of COX. As cyanide showed a significantly stronger inhibition than azide (see S3 Fig. Propylgallate Azide and S4 Fig. Propylgallate Cyanide) it was used for the following assays.

For the inhibition of the AOX we compared the effect of three different inhibitors reported in literature [29, 36, 37]: SHAM, BHAM and *n*-propyl gallate (n-PG). A stable inhibition with SHAM was reached after approximately 15 minutes of exposure (Fig 2c and 2e). The hydroxamic acid BHAM showed a similar time dependency but the inhibition was 45% less compared to SHAM (see S2 Fig. BHAM vs. SHAM). In contrast, the chemically different and more potent inhibitor n-PG [38] caused an immediate and sharp decline in oxygen consumption rates. However, also with this inhibitor, the level of inhibition was lower compared to SHAM (see S2 Fig. BHAM vs. SHAM and S4 Fig. Propylgallate Cyanide). Considering the lower inhibition of AOX activity by BHAM and n-PG we used SHAM for further experiments.

The protonophores CCCP, FCCP and DNP were compared for uncoupling ETS and thus stimulating oxygen consumption. FCCP and CCCP caused a similar increase in oxygen consumption rates, but with different patterns. FCCP stimulated the oxygen consumption rate but always inhibited oxygen consumption rate above a certain concentration. This was sometimes also observed with CCCP. In contrast, DNP neither stimulated nor inhibited the oxygen consumption rate. Finally, CCCP was chosen and used throughout our study because it showed less interference at levels above optimum concentration.

Sequence of respirometric assays. Considering the above mentioned time dependency (i) of the inhibitory effect of SHAM as a result of permeation limitations and the (ii) restricted availability of oxygen in a closed chamber system, we established a standardised sequence of six single measurements in three respirometric assays (Fig 2). Keeping the single measurements short was also necessary because a re-oxygenation (i.e. aeration of the chamber after the oxygen concentration has already reached a critical level) was not possible with *P*. *ochrochloron*: After re-oxygenation, the initial level of oxygen consumption rate was not fully established, and moreover, the oxygen consumption signal started to get unstable. Both observations indicated a physiological adaptation and therefore a continuation of measurements was not advisable.

The standardised sequence consisted of three independent assays (Fig 2). In the first assay (Fig 2a and 2b) the stability of the oxygen consumption rate and the influence of the solvent used for inhibitor solubilisation were checked. For estimation of the maximal uncoupling capacity the proton gradient across the inner mitochondrial membrane was collapsed with the protonophore CCCP. In the second assay (Fig 2c and 2d) the inhibition of both terminal oxidases was performed. This was done also with a *vice versa* sequence of inhibitors. Thirdly, after further 24 hours of chemostat cultivation, the second assay (Fig 2e and 2f) was repeated. This inter-day validation served for validating the reproducibility and for monitoring the stability of steady state cultures.

### 3.2 Applying the optimized methods for cultivation, sample preparation and respirometry

**Fig 3:**
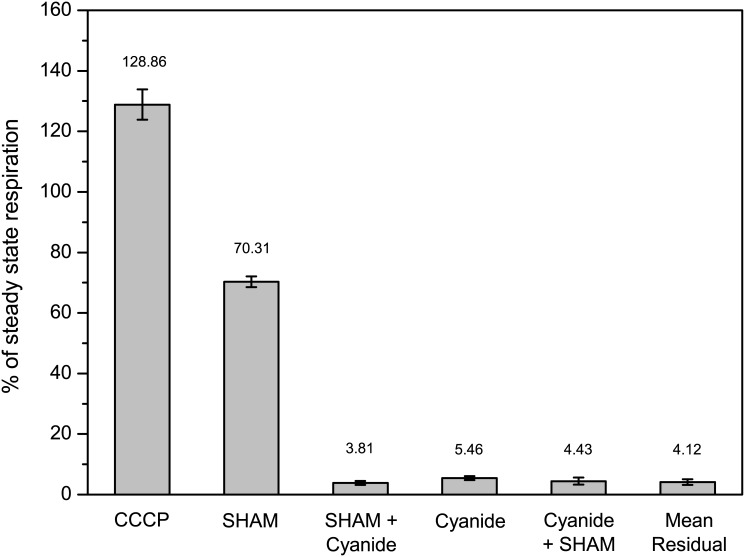
Oxygen consumption of glucose-limited steady state mycelium of *Penicillium ochrochloron*. Oxygen consumption in the presence of SHAM (AOX inhibitior), cyanide (COX inhibitior) and CCCP (uncoupler of mitochondrial proton gradient). The oxygen consumption rates were normalized to the steady state oxygen consumption rate without inhibitors. The bars represent mean and standard deviation of three separate steady state cultivations (see S1 Table Off-line respirometry). AOX was inhibited with 2.5 mM SHAM, COX with 1 mM cyanide, and uncoupling was done with 3 μM CCCP. The mean residual oxygen consumption was calculated with both inhibitors present.

Combining the optimized procedures for cultivation, sample preparation and the respirometric assay, resulted in the data set (S1 Table Off-line respirometry) depicted in Fig 3. Uncoupling the ETS with CCCP revealed that glucose-limited steady state mycelium used only about two thirds of its maximal respiratory capacity (Fig 3). Inhibiting the activity of AOX with SHAM decreased the oxygen consumption rate about one third within 15 minutes (Fig 3). A subsequent inhibition of the COX with cyanide further diminished the remaining oxygen consumption to approximately 5% (Fig 3).

In contrast, if the sequence of inhibitors was reversed and the sequence started with cyanide, the oxygen consumption collapsed almost completely (Fig 3). A subsequent application of SHAM caused only a very slight decrease of the oxygen consumption rate (Fig 3). However, the total rate of inhibition with both inhibitors present was independent of the applied sequence and the residual oxygen consumption in glucose-limited steady state mycelium was about 5% (Fig 3).

## Discussion

Many questions concerning fungal respiratory systems are still unanswered. One question was pointed out by Joseph-Horne et al. [13] in their comprehensive review as follows: ‘One of the key questions for fungal AOX is whether it is constitutively expressed, and if so, is it an active component of the electron transport chain’. Unfortunately, the answer is not straightforward. Constitutive expression of AOX was reported in *A*. *niger* [39] and in *N*. *crassa* [3], though the latter authors could not find any corresponding enzymatic activity. Another study with *N*. *crassa* suggested that the AOX is inducible by several factors [40].

There is another fundamental open question: In fungi it is yet unclear, if the AOX serves merely as an ‘electron sink’ to mediate an electron overflow in case the cytochrome pathway is saturated or blocked, or, if this alternative pathway might ‘compete with an *un*saturated cytochrome pathway’ as it was suggested for plants [41].

### 4.1 Phenotypic plasticity, standardisation and reference mycelium

Currently there is no standard protocol to study fungal ETS *in vivo*–neither for growth conditions nor for sample preparation nor for the respirometric assay. A wide range of conditions and methods were reported for functionally exploring fungal ETS (Table 1).

In “-omics” studies it is state of the art that cultivation, sampling, and physiological assay conditions of microorganisms must be adapted to each species, each strain, and even to each physiological phenotype anew. This is also true for studying the ETS and is especially important with filamentous fungi with their high phenotypic plasticity. The physiological phenotype of filamentous fungi is highly susceptible to the kind of nutrient limitation and small changes in all kinds of experimental margin conditions [33]. Because of this, it is neither reasonable nor possible to develop one single standard protocol to study the ETS in different fungal organisms. Exactly because of this situation it is all the more important to standardise cultivation methods, sampling procedures and assay conditions as extensive as necessary.

Thus the first prerequisite was to choose a cultivation method which leads to a reproducible and defined physiological state. Obviously, only chemostat cultivation could be the method of choice. We used glucose as the limiting nutrient for three reasons: (i) Glucose-limited chemostat mycelium of *P*. *ochrochloron* was already characterized on several physiological levels, e. g. glucose uptake [33]. (ii) With glucose-limited chemostat mycelium a stable steady state respiration over a period of 20 minutes was achieved. And (iii) glucose limitation resulted in only a negligible degree of organic acid excretion [42]. If the AOX plays a role during organic acid excretion, then AOX activity should be lowered during non-excreting growth conditions. Respirometric data from glucose-limited chemostat mycelium thus provide an important reference point to compare chemostat mycelia from different nutrient limitations.

Second, a further aim was to expose the mycelium to as little manipulations as possible during transfer from chemostat to the respirometer, i. e. during sample preparation. This was achieved by transferring a few drops of culture broth directly from the chemostat to the respirometer. This guaranteed that the physiological state of the hyphae in the respirometer was as close as possible to that during steady state growth in the chemostat.

And the third level of standardisation was to establish a standardised assay protocol to test the effect of inhibitors and uncouplers on oxygen consumption rate in the high-resolution respirometer.

### 4.2 Applying high-resolution respirometry to glucose-limited steady state mycelium of *P*. *ochrochloron*

The tested inhibitors and uncouplers varied considerably in their effectivity. While some, like DNP, did not show any effect on oxygen consumption rate, the effect of others, like SHAM, was clearly time dependent (Fig 2c and 2e). This indicated a limitation in the permeation of the combined barrier of cell wall and plasma membrane. It should also be kept in mind that anti-drug pumps may remove inhibitors from the cytoplasm [16].

The oxygen consumption of glucose-limited steady state mycelium of *P*. *ochrochloron*, i.e. hyphae which excrete no significant amounts of organic acids, showed several distinct characteristics:

1. The uncoupling of ETS indicated that the oxygen consumption rate of *P*. *ochrochloron* was distinctly below the maximum respiratory capacity (Fig 3). This observation is consistent with previous findings with *P*. *ochrochloron*, where the oxygen consumption increased immediately after a glucose pulse to glucose-limited chemostat mycelium [33].
2. Like in the industrial strain *A*. *niger* B60, in *P*. *ochrochloron* a rotenone-sensitive complex I was active under conditions with no or negligible organic acid excretion. In *A*. *niger* B60 the activity of complex I got selectively lost with the onset of organic acid production [10], while activity of alternative NADH-dehydrogenases was enhanced [2, 10]. Whether this is also the case in *P*. *ochrochloron* yet needs to be answered.
3. Although it is likely that *P*. *ochrochloron* possesses a complex III like other fungi [13], we were unable to demonstrate its inhibition, since 20 μM antimycin A did not show any effect on the oxygen consumption rate. However, in the light of the difficulties with a series of inhibitors and uncouplers (see section Results), the most probable explanation is that antimycin A could not pass the barrier of cell wall and plasma membrane under the tested conditions.
4. Similar to other fungi [13], we found a cyanide sensitive COX (complex IV) in *P*. *ochrochloron* under glucose-limited conditions.
5. Whether or not AOX was active depends on the point of view. Commonly, the respiration attributed to AOX is defined as respiratory activity which is insensitive to cyanide and sensitive to SHAM, whereby the sequence of added inhibitors is of relevance (i.e. cyanide prior to SHAM). Following this sequence of inhibitor addition, the AOX seemed not to be present in *P ochrochloron* under glucose-limited conditions (Fig 3). In contrast, if the sequence of inhibitors was reversed, i.e. SHAM prior to cyanide, AOX attributed with approximately 30% to the total respiration. As listed in Table 2, in other studies it was also reported that the value of AOX inhibition depended on the sequence of inhibitor addition. Section 4.3 will address this issue in more detail.
6. If both terminal oxidases were fully inhibited, the residual oxygen consumption was—irrespective of the sequence of inhibitor addition—about 5% of the steady state oxygen consumption rate (Fig 3). This value is within the range reported for residual respiratory activity in filamentous fungi (Table 2). The nature of this respiration remains speculative, but it might be due to oxygen requiring processes like leak respiration or generation of reactive oxygen species. Another demand for molecular oxygen might stem from non-respiratory anabolic pathways as stated for unicellular fungi [43].

**Table 2:** Compilation of studies dealing with the ETS of filamentous fungi applying SHAM or BHAM ^b^ for AOX and cyanide for COX or Antimycin A for complex III. The kind of used cells, the degree of inhibition, residual respiration and sequence of inhibitor addition are given. n.d., not determined; perm., permeabilised with digitonin;

Organism	Sample	COX or complex III inhibited prior to AOX Relative contribution to steady state respiration in %			AOX inhibited prior to COX or complex III Relative contribution to steady state respiration in %			Reference
		COX	AOX	Residual	COX	AOX	Residual	
*Aspergillus fumigatus* wt	perm. spheroplasts	78	17 ^b^	5	81	15 ^b^	4	[36]
*Aspergillus niger* B 60	whole mycelium	n.d.	n.d.	n.d.	75	12	13	[7]
*Aspergillus niger* B 60	whole mycelium	22 ^a^	67	11	75 ^a^	13	12	[7]
*Aspergillus niger* WU-2223L	whole mycelium	88	12	0	34	66	0	[25]
*Aspergillus niger* WU-2223L AOXEGFP-1	single cell conidia	73	27	0	94	6	0	[39]
*Metarhizium anisopliae* ARSEF 2083	whole mycelium	20	53	27	68	7	25	[28]
*Neurospora crassa* NCN 235	whole mycelium	80	13	7	90	0	10	[28]
*Phycomyces blakesleeanus* NRRL 1555	whole mycelium	73	27	0	73	27	0	[24]
*Penicillium ochrochloron* CBS 123.824	whole mycelium	95	1	4	66	30	4	Present work

^a^ Antimycin A for inhibiting complex III;

^b^ BHAM for inhibiting AOX;

### 4.3 Activity of AOX in dependence of the inhibitor sequence

**Fig 4:**
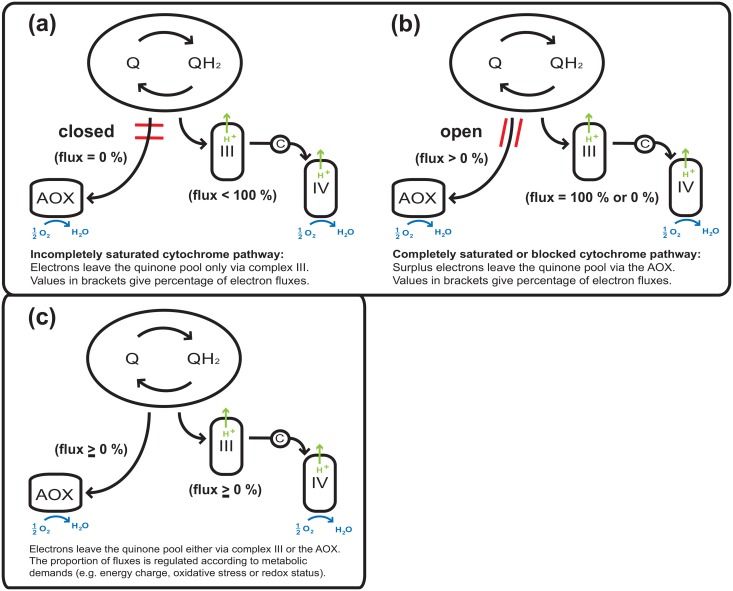
Hypothesized electron partitioning between COX and AOX. The safety valve hypothesis suggests two distinct states: (a) Electrons are not drained to AOX if the cytochrome pathway is not operating to full extent. (b) Electrons flow to AOX in situations of a fully saturated or blocked cytochrome pathway. (c) The dynamic inter-dependence hypothesis in contrast excludes a regulation of AOX activity solely by the degree of saturation or a blockage of the cytochrome pathway. Electron partitioning is regulated dynamically by different metabolic demands.

As mentioned before, AOX activity is generally considered as the respiration which is insensitive to cyanide and sensitive to SHAM. This viewpoint is reflected in the usual sequence of added inhibitors: First a specific inhibitor for the COX, like cyanide, is applied, followed by a specific inhibitor of the AOX. The decrease in respiration after this second step is usually attributed to the activity of the AOX. However, as indicated above, a reversal of this sequence of inhibitors can result in significant different values of AOX inhibition (examples for other filamentous fungi see Table 2)–an aspect which has been noted by others before [25, 29]. If we assume that this is not an experimental artifact from non-specific side-effects of the inhibitor, then these observations shade light on the fundamental question whether or not the AOX serves as a safety overflow valve for a fully saturated or blocked cytochrome pathway (Fig 4a and 4b). The fact that the sequence of inhibitor addition strongly determined the degree of inhibition by SHAM in *P*. *ochrochloron* and other filamentous fungi (Table 2) suggests that AOX serves—similar to plants—not as a safety valve. Activity of AOX should rather be viewed as being in a dynamic interdependence with the cytochrome pathway (Fig 4c) [41, 44]. As the electron fluxes to both oxidases might be interconnected dynamically via the quinone pool, the results from inhibitor experiments do not necessary reflect the activity of COX and AOX *in vivo*. The methods required to determine the *in vivo* contribution of each pathway to the respiratory activity have yet to be developed for filamentous fungi. Due to their tremendous phenotypic plasticity, it is probable that these methods have to be adapted not only to each organism but also to each physiological condition anew.

## Supporting Information

S1 Appendix Sample preparation. (DOCX)

S2 Appendix Biomass. (DOCX)

S1 Table Off-line respirometry. (XLSX)

S2 Table On-line respirometry. (XLSX)

S1 Fig Glucose supplement. (DOCX)

S2 Fig BHAM vs. SHAM. (DOCX)

S3 Fig Propylgallate Azide. (DOCX)

S4 Fig Propylgallate Cyanide. (DOCX)

S5 Fig Rotenone Antimycin A. (DOCX)

## Abbreviations

AOX: alternative oxidase
BHAM: benzhydroxamic acid
CCCP: carbonyl cyanide *m*-chlorophenylhydrazone
COX: cytochrome c oxidase
DNP: 2,4-dinitrophenol
ETS: electron transport system
FCCP: carbonyl cyanide 4-(trifluoromethoxy) phenylhydrazone
HEPES: N-2-hydroxyethylpiperazine-N′-2-ethanesulfonic acid
SHAM: salicylhydroxamic acid

## References

[1] Carneiro P, Duarte M, Videira A. The external alternative NAD(P)H dehydrogenase NDE3 is localized both in the mitochondria and in the cytoplasm of Neurospora crassa. J Mol Biol. 2007;368:1114–1121. 17379240 10.1016/j.jmb.2007.02.080

[2] Prömper C, Schneider R, Weiss H. The role of the proton pumping and alternative respiratory chain NADH Ubiquinone Oxidoreductases in overflow catabolism of *Aspergillus niger*. Eur J Biochem. 1993; 216:223–230. 8365409 10.1111/j.1432-1033.1993.tb18136.x

[3] Tanton LL, Nargang CE, Kessler KE, Li QH, Nargang FE. Alternative oxidase expression in Neurospora crassa. Fung Genet Biol. 2003;39: 176–190. 10.1016/s1087-1845(03)00002-1 12781676

[4] Magnani T, Soriani FM, Martins VdP, de Freitas Policarpo AC, Sorgi CA, Faccioli LH, et al Silencing of mitochondrial alternative oxidase gene of *Aspergillus fumigatus* enhances reactive oxygen species production and killing of the fungus by macrophages. J Bioenerget Biomemb. 2008; 40:631–636. 10.1007/s10863-008-9191-5 19148712

[5] Grahl N, Dinamarco TM, Willger SD, Goldman GH, Cramer RA. *Aspergillus fumigatus* mitochondrial electron transport chain mediates oxidative stress homeostasis, hypoxia responses and fungal pathogenesis. Mol Microbiol. 2012; 84:383–899. 10.1111/j.1365-2958.2012.08034.x 22443190 PMC3323727

[6] Kozma J, Karaffa L. Effect of oxygen on the respiratory system and cephalosporin-C production in *Acremonium chrysogenum*. J Biotechnol. 1996; 48:59–66. 8818273 10.1016/0168-1656(96)01400-9

[7] Zehentgruber O, Kubicek CP, Rohr M. Alternative respiration of *Aspergillus niger*. Fems Microbiol Lett. 1980; 8:71–74.

[8] Kirimura K, Yoda M, Shimizu H, Sugano S, Mizuno M, Kino K, et al Contribution of cyanide-insensitive respiratory pathway, catalyzed by the alternative oxidase, to citric acid production in *Aspergillus niger*. Biosci Biotechnol Biochem. 2000; 64:2034–2039. 11129572 10.1271/bbb.64.2034

[9] Kubicek CP, Zehentgruber O, Elkalak H, Rohr M. Regulation of citric acid production by oxygen—effect of dissolved oxygen tension on adenylate levels and respiration in *Aspergillus niger*. Europ J Appl Microbiol Biotechnol. 1980; 9:101–115.

[10] Wallrath J, Schmidt M, Weiss H. Concomitant loss of respiratory chain NADH Ubiquinone Reductase (Complex I) and citric acid accumulation in Aspergillus niger. Appl Microbiol Biotechnol. 1991; 36:76–81.

[11] Joseph-Horne T, Hollomon DW. Functional diversity within the mitochondrial electron transport chain of plant pathogenic fungi. Pest Manag Sci. 2000;56: 24–30.

[12] Martins VP, Dinamarco TM, Soriani FM, Tudella VG, Oliveira SC, Goldman GH, et al Involvement of an Alternative Oxidase in Oxidative Stress and Mycelium-to-Yeast Differentiation in *Paracoccidioides brasiliensis*. Eukaryotic Cell. 2011; 10:237–248. 10.1128/EC.00194-10 21183691 PMC3067407

[13] Joseph-Horne T, Hollomon DW, Wood PM. Fungal respiration: a fusion of standard and alternative components. Biochim Biophys Acta—Bioenergetics. 2001; 1504:179–195. 10.1016/s0005-2728(00)00251-6 11245784

[14] Nargang FE, Kennell JC. Mitochondria and respiration In: Borkovich KA, Ebbole DE, editors. Cellular and molecular biology of filamentous fungi. Washington DC: ASM Press; 2010 p. 155–178.

[15] Griffin DH. Fungal physiology. 2nd ed New York: Wiley-Liss; 1994 p. 159.

[16] Costa C, Dias PJ, Sa-Correia I, Teixeira MC. MFS multidrug transporters in pathogenic fungi: do they have real clinical impact? Frontiers in Physiology. 2014; 5:197 10.3389/fphys.2014.00197 24904431 PMC4035561

[17] Foster JW. Chemical activities of fungi. New York: Academic Press; 1949.

[18] Slepecky RA, Starmer WT. Phenotypic plasticity in fungi: a review with observations on Aureobasidium pullulans. Mycologia. 2009;101(6):823–32. 19927747 10.3852/08-197

[19] van Gulik WM, Canelas AB, Seifar RM, Heijnen JJ. The Sampling and Sample Preparation Problem in Microbial Metabolomics Metabolomics in Practice: Wiley-VCH Verlag GmbH & Co. KGaA; 2013 p. 1–19.

[20] Nielsen J, Jewett MC, editors, Metabolomics A powerful tool in systems biology Topics in Current Genetics Series. Heidelberg: Springer; 2007 p. 1–10. 10.1007/978-3-540-74719-2

[21] de Jonge LP, Douma RD, Heijnen JJ, van Gulik WM. Optimization of cold methanol quenching for quantitative metabolomics of Penicillium chrysogenum. Metabolomics. 2012;8(4):727–35. 22833711 10.1007/s11306-011-0367-3 PMC3397231

[22] Villas-Boas SG, Hojer-Pedersen J, Akesson M, Smedsgaard J, Nielsen J. Global metabolite analysis of yeast: evaluation of sample preparation methods. Yeast. 2005; 22(14):1155–69. 16240456 10.1002/yea.1308

[23] Egli T. Microbial growth and physiology: a call for better craftsmanship. Frontiers in Microbiology. 2015; 6:287 10.3389/fmicb.2015.00287 25926822 PMC4396425

[24] Zivic M, Zakrzewska J, Stanic M, Cvetic T, Zivanovic B. Alternative respiration of fungus *Phycomyces blakesleeanus*. Antonie Van Leeuwenhoek. 2009; 95:207–217. 10.1007/s10482-008-9304-5 19125346

[25] Kirimura K, Hirowatari Y, Usami S. Alterations of respiratory systems in Aspergillus niger under the conditions of citric acid fermentation. Agric Biol Chem. 1987; 51:1299–1303.

[26] Kirimura K, Matsui T, Sugano S, Usami S. Enhancement and repression of cyanide-insensitive respiration in *Aspergillus niger*. Fems Microbiol Lett. 1996; 141:251–254. 8768530 10.1111/j.1574-6968.1996.tb08393.x

[27] Yukioka H, Tanaka R, Inagaki S, Katoh K, Miki N, Mizutani A, et al Mutants of the phytopathogenic fungus Magnaporthe grisea deficient in alternative, cyanide-resistant, respiration. Fung Genet Biol. 1997; 22:221–228. 10.1006/fgbi.1997.1016 9454649

[28] Uribe D, Khachatourians GG. Identification and characterization of an alternative oxidase in the entomopathogenic fungus *Metarhizium anisopliae*. Can J Microbiol. 2008; 54:119–127. 10.1139/w07-127 18388981

[29] Lambowitz AM, Slayman CW. Cyanide resistant respiration in *Neurospora crassa*. J Bacteriol. 1971;108:1087–1096. 4333318 10.1128/jb.108.3.1087-1096.1971 PMC247191

[30] Karaffa L, Sandor E, Kozma J, Szentirmai A. Cephalosporin-C production, morphology and alternative respiration of a *Acremonium chrysogenum* in glucose-limited chemostat. Biotechnol Lett. 1996; 18:701–706.

[31] Bai ZH, Harvey LM, White S, McNeil B. Effects of oxidative stress on production of heterologous and native protein, and culture morphology in batch and chemostat cultures of Aspergillus niger (B1-D). Enzyme Microb Technol. 2004; 34:10–21.

[32] Franz A, Burgstaller W, Schinner F. Leaching with *Penicillium simplicissimum*–influence of metals and buffers on proton extrusion and citric acid production. Appl Environ Microbiol. 1991; 57:769–774. 16348442 10.1128/aem.57.3.769-774.1991 PMC182793

[33] Vrabl P, Mutschlechner W, Burgstaller W. Characteristics of glucose uptake by glucose- and NH4-limited grown Penicillium ochrochloron at low, medium and high glucose concentration. Fung Genet Biol. 2008; 45:1380–1392. 10.1016/j.fgb.2008.07.017 18722543

[34] Ganzera M, Vrabl P, Woerle E, Burgstaller W, Stuppner H. Determination of adenine and pyridine nucleotides in glucose-limited chemostat cultures of *Penicillium simplicissimum* by one-step ethanol extraction and ion-pairing liquid chromatography. Anal Biochem. 2006; 359:132–140. 17054897 10.1016/j.ab.2006.09.012

[35] Gibbs PA, Seviour RJ, Schmid F. Growth of filamentous fungi in submerged culture: Problems and possible solutions. Crit Rev Biotechnol. 2000; 20:17–48. 10770226 10.1080/07388550091144177

[36] Tudella VG, Curti C, Soriani FM, Santos AC, Uyemura SA. In situ evidence of an alternative oxidase and an uncoupling protein in the respiratory chain of *Aspergillus fumigatus*. Int J Biochem Cell Biol. 2004; 36:162–172. 14592541 10.1016/s1357-2725(03)00194-8

[37] Parrish DJ, Leopold AC. Confounding of alternative respiration by lipoxygenase activity. Plant Physiol. 1978; 62: 470–472. 16660540 10.1104/pp.62.3.470 PMC1092149

[38] Siedow JN, Girvin ME. Alternative respiratory pathway—its role in seed respiration and its inhibition by propyl gallate. Plant Physiol. 1980; 65:669–674. 16661259 10.1104/pp.65.4.669 PMC440403

[39] Hattori T, Honda Y, Kino K, Kirimura K. Expression of alternative oxidase gene (aox1) at the stage of single-cell conidium in citric acid-producing Aspergillus niger. J Biosci Bioeng. 2008; 105:55–57. 10.1263/jbb.105.55 18295720

[40] Descheneau AT, Cleary IA, Nargang FE. Genetic evidence for a regulatory pathway controlling alternative oxidase production in *Neurospora crassa*. Genetics. 2005;169:123–135. 15466423 10.1534/genetics.104.034017 PMC1448880

[41] Sluse FE, Jarmuszkiewicz W. Alternative oxidase in the branched mitochondrial respiratory network: an overview on structure, function, regulation, and role. Braz J Med Biol Res. 1998; 31:733–747. 9698817 10.1590/s0100-879x1998000600003

[42] Gallmetzer M, Burgstaller W. Citrate efflux in glucose-limited and glucose-sufficient chemostat culture of *Penicillium simplicissium*. Antonie Van Leeuwenhoek. 2001; 79:81–87. 11392488 10.1023/a:1010295924549

[43] Rosenfeld E, Beauvoit B. Role of the non-respiratory pathways in the utilization of molecular oxygen by *Saccharomyces cerevisiae*. Yeast. 2003; 20:1115–1144. 14558145 10.1002/yea.1026

[44] Siedow JN, Umbach AL. The mitochondrial cyanide-resistant oxidase: structural conservation amid regulatory diversity. Biochim Biophys Acta-Bioenerg. 2000; 1459(2–3):432–9. 10.1016/s0005-2728(00)00181-x 11004460

