# Supplementary material for: Adapting High-Resolution Respirometry to Glucose-Limited Steady State Mycelium of the Filamentous Fungus Penicillium ochrochloron: Method Development and Standardisation
Source: PLoS One. 2016 Jan 15;11(1):e0146878. doi: 10.1371/journal.pone.0146878 (PMC4714917; doi:10.1371/journal.pone.0146878)
Supplement: S1 Appendix — (DOCX) [file pone.0146878.s001.docx]

Supplementary Material to the original research article in the journal “PLOS ONE”:

Adapting high-resolution respirometry to glucose-limited steady state mycelium of the filamentous fungus *Penicillium ochrochloron*:

Method development and standardisation

Christoph W. Schinagl*, Pamela Vrabl and Wolfgang Burgstaller

University of Innsbruck, Institute of Microbiology, Technikerstrasse 25, 6020 Innsbruck, Austria

* CORRESPONDING AUTHOR: Christoph W. Schinagl, University of Innsbruck, Institute of Microbiology, Technikerstrasse 25, A-6020 Innsbruck, Austria

Email: [christoph.schinagl@uibk.ac.at](mailto:christoph.schinagl@uibk.ac.at)

**S1 Appendix Sample preparation**

**Development of sample handling for high-resolution respirometry**

Starting point was the separation of biomass from the culture broth by filtration. All tested variations (a-c) failed and therefore filtration was ruled out because of its negative interference on respiration. Finally, a direct transfer of cultivation broth (d) paved the way for reproducibility.


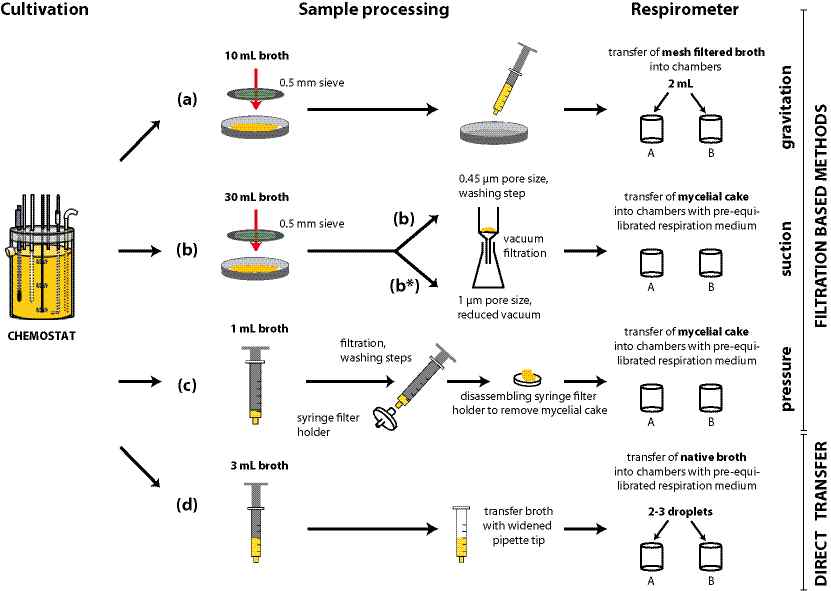


Fig A Different sample preparations tested in the course of the method development. In d (direct transfer) the final standard workflow for sample handling in this study is depicted.

**Gravimetric filtration (a):** A volume of 10 mL culture broth was withdrawn from the bioreactor and filtered by gravitation through a stainless steel sieve (mesh size 0.5 mm). 2 mL of the resulting filtrate, which contained a very homogenous mycelium, was transferred in each chamber of the respirometer.

**Filtration by vacuum (b):** To increase the biomass concentration in the assay and thus the oxygen consumption signal, the sample volume withdrawn from the bioreactor was increased to approximately 20-30 mL. The sample was then gravimetrically filtered in a first step as described above. In a next step the resulting filtrate was additionally vacuum filtered (cellulose acetate, pore size 0.45 µm; filter cake always remained moist) and washed with 10 mL pre-warmed (30 °C) respiration medium (Fig 1 b). As this did not result in the targeted stable respiration, this procedure was slightly modified by reducing the applied vacuum in the filtration step to maximal 250 mbar relative to air pressure and using a nylon net with a pore size of 1 µm as filter (b*).

**Filtration by pressure (c):** With a syringe 1 mL culture broth was withdrawn from the bioreactor and passed through a paper filter (Ederol No. 15) which was placed in a Sartorius syringe filter holder. Before transferring the biomass to the respirometer and starting the assay, the filter cake in the syringe holder was washed with 5 mL pre-warmed (30 °C) respiration medium.

**Direct transfer (d):** With a syringe 3 mL culture broth was withdrawn from the bioreactor. Than immediately 2-3 droplets where directly transferred with a 1 mL piston pipette into each chamber of the respirometer, where 2 mL of respiration medium had been already pre-equilibrated. Due to the fungal morphology it was necessary to widen the tip pore of the pipette with a capillary cutter. A widening of the tip pore to 2 mm inner diameter enabled a more reproducible transfer of mycelium.
