# Supplementary material for: Adapting High-Resolution Respirometry to Glucose-Limited Steady State Mycelium of the Filamentous Fungus Penicillium ochrochloron: Method Development and Standardisation
Source: PLoS One. 2016 Jan 15;11(1):e0146878. doi: 10.1371/journal.pone.0146878 (PMC4714917; doi:10.1371/journal.pone.0146878)
Supplement: S2 Appendix — (DOCX) [file pone.0146878.s002.docx]

Supplementary Material to the original research article in the journal “PLOS ONE”:

Adapting high-resolution respirometry to glucose-limited steady state mycelium of the filamentous fungus *Penicillium ochrochloron*:

Method development and standardisation

Christoph W. Schinagl*, Pamela Vrabl and Wolfgang Burgstaller

University of Innsbruck, Institute of Microbiology, Technikerstrasse 25, 6020 Innsbruck, Austria

* CORRESPONDING AUTHOR: Christoph W. Schinagl, University of Innsbruck, Institute of Microbiology, Technikerstrasse 25, A-6020 Innsbruck, Austria

Email: [christoph.schinagl@uibk.ac.at](mailto:christoph.schinagl@uibk.ac.at)

**S2 Appendix Biomass**

**Collecting biomass from the respirometer chambers:**

All attempts such as carefully rinsing chambers of the respirometer, capillaries and outer surfaces of the stoppers to quantitatively transfer the entire biomass, and a subsequent centrifugation of the resulting liquids and/or filtration failed to deliver reliable and reproducible results for dry weight of biomass. The reason for this problem was probably caused by the chemical treatment of the mycelium during the assay. Another attempt to estimate biomass as dry weight by applying the same volume of sample as for the assay into test tubes and evaporating the fluid was compromised by salts remaining from the chemostat media.

**Calculation of biomass in the assay from data of chemostat culture:**

- We converted the oxygen consumption rate from the on-line respirometer of the bioreactor ((mL O_2_) min^-1^) to mol sec^-1^ mL^-1^, the units given by the Oxygraph 2K for the oxygen consumption rate.
- 4.24 ±2 % (mL O_2_) min^-1^ then give 1.6 x 10^-9^ mol sec^-1^ mL^-1^ (using the molar gas volume at 30 °C, i. e. 24.88 L mol^-1^).
- On average, the steady state oxygen consumption rate measured in the Oxygraph was 6 x 10^-11^ mol sec^-1^ mL^-1^.
- Thus giving a factor of 27 between oxygen consumption rates from the Oxygraph 2k and chemostat.

Many cultivation experiments in bioreactors coupled to on-line respirometry confirmed that the oxygen consumption rate is strictly correlated to the amount of biomass.

- On average the biomass concentration in the chemostat was 3.62 ± 10 % (g DW) L^-1^ or 3.62 (mg DW) mL^-1^.
- Applying the calculated factor of 27 to the measured biomass concentration of 3.62 (mg DW) mL^-1^ gives 0.134 (mg DW) mL^-1^ or 0.27 (mg DW) (2 mL)^-1^, i. e. per chamber.
