# Supplementary material for: Adapting High-Resolution Respirometry to Glucose-Limited Steady State Mycelium of the Filamentous Fungus Penicillium ochrochloron: Method Development and Standardisation
Source: PLoS One. 2016 Jan 15;11(1):e0146878. doi: 10.1371/journal.pone.0146878 (PMC4714917; doi:10.1371/journal.pone.0146878)
Supplement: S1 Fig — (DOCX) [file pone.0146878.s005.docx]

**S1 Fig. Glucose supplement**

S1 Fig. Simultaneously measurement of mycelium from a steady state sample, chamber A (upper panel) and chamber B (lower panel). Chamber A: stability of respiratory flux in respiration medium supplemented with 1 mM glucose. Chamber B: Stability of respiratory flux in respiration medium without any supplementation. Allthough both chambers were loaded with three droplets of culture broth, the respiratory flux in chamber A with glucose supplementation is highly elevated compared to chamber B. Stability of respiratory flux in chamber B is as defined in section 2.6.
