# Supplementary material for: Adapting High-Resolution Respirometry to Glucose-Limited Steady State Mycelium of the Filamentous Fungus Penicillium ochrochloron: Method Development and Standardisation
Source: PLoS One. 2016 Jan 15;11(1):e0146878. doi: 10.1371/journal.pone.0146878 (PMC4714917; doi:10.1371/journal.pone.0146878)
Supplement: S4 Fig — (DOCX) [file pone.0146878.s008.docx]

**S4 Fig. Propylgallate Cyanide**

S4 Fig. Propylgallate Cyanide. Simultaneously measurement of mycelium from a steady state sample. Chamber A (upper panel) and chamber B (lower panel). Chamber A: n-PG followed by cyanide, chamber B: *vice versa* sequence of inhibitors.
